# Supplementary material for: Frequency-Dependent Dielectric Permittivity and Water Permeability in Ordered Mesoporous Silica-Grafted Fluorinated Polyimides
Source: Polymers (Basel). 2024 Jun 16;16(12):1716. doi: 10.3390/polym16121716 (PMC11207492; doi:10.3390/polym16121716)
Supplement: Supplementary file 1 [file polymers-16-01716-s001.zip › polymers-3001692-supplementary.pdf]

Supplementary Materials for

Frequency-Dependent Dielectric Permittivity and Water Permeability in Ordered Mesoporous Silica-Grafted Fluorinated Polyimides

*Jaemin Son, Hwon Park, Minju Kim, Jae Hui Park, Ki-Ho Nam * and Jin-Seok Bae **

Department of Textile System Engineering, Kyungpook National University, Daegu 41566, Republic of Korea

Corresponding author. E-mail: knam@knu.ac.kr, jbae@knu.ac.kr

Table of Contents

A. Thermal and thermooxidative behaviors of the FPI-g-AMS films.
B. Dielectric constant (Dk) and dissipation factor (Df) of the FPI-g-AMS films at a frequency of 1 MHz.
C. Water barrier properties of the FPI-g-AMS films.

Supplementary Tables

Analysis of thermal properties of the FPI-*g*-AMS films

Supplementary Table S1. Thermal and thermooxidative behaviors of the FPI-g-AMS films

Sample code	T_g (°C)	$T_{5\%}$ (°C) [a]	Char yield (%) [b]
FPI-*g*-AMS-0	302	539	57
FPI-*g*-AMS-0.5	301	536	56
FPI-*g*-AMS-1	302	532	55
FPI-*g*-AMS-3	301	531	54
FPI-*g*-AMS-6	300	534	51

[a]Decomposition temperature at 5% weight loss. [b]Weight percentage of char residues at 800 °C.

Analysis of dielectric permittivity of the FPI-*g*-AMS films

Supplementary Table S2. Dielectric constant (D_k) and dissipation factor (D_f) of the FPI-g-AMS films at a frequency of 1 MHz

Sample code	D_k	D_f
FPI-*g*-AMS-0	2.91	0.00437
FPI-*g*-AMS-0.5	2.86	0.00363
FPI-*g*-AMS-1	2.76	0.00314
FPI-*g*-AMS-3	2.69	0.00264
FPI-*g*-AMS-6	2.67	0.00369

Analysis of water barrier properties of the FPI-*g*-AMS films

Supplementary Table S3. Water barrier properties of the FPI-g-AMS films

Sample code	Permeability ($\times 10^{-8}$ mol s^{-1} m^{-1} atm^{-1})	Diffusivity ($\times 10^{-12}$ m^2 s^{-1})	Solubility ($\times 10^4$ mol m^{-3} atm^{-1})	Contact angle (°)
FPI-*g*-AMS-0	1.52	1.10	1.38	85.2
FPI-*g*-AMS-0.5	1.44	1.09	1.31	91.2
FPI-*g*-AMS-1	1.19	1.05	1.13	91.3
FPI-*g*-AMS-3	1.10	1.07	1.03	90.3
FPI-*g*-AMS-6	1.26	1.09	1.16	87.4
